# Iron Deficiency and Internalizing Symptoms Among Adolescents in the National Health and Nutrition Examination Survey

**DOI:** 10.3390/nu16213643

**Published:** 2024-10-26

**Authors:** Dimitri Fiani, Solangia Engler, Yang Ni, Sherecce Fields, Chadi Calarge

**Affiliations:** 1 Department of Psychiatry and Psychology, Neurological Institute, Cleveland Clinic, Cleveland, OH 44195, USA; 2 Department of Psychological & Brain Sciences, Texas A&M University, College Station, TX 77843, USA; 3 Department of Statistics, Texas A&M University, College Station, TX 77843, USA; 4 Menninger Department of Psychiatry and Behavioral Sciences, Department of Pediatrics, Baylor College of Medicine, Houston, TX 77030, USA

**Keywords:** iron deficiency, depression, anxiety, adolescents

## Abstract

Background: Iron Deficiency (ID) affects two billion people worldwide, predominantly adolescent girls, and may be associated with increased psychopathology. The associations between ID and symptoms of depression and anxiety in adolescents were examined using data from the National Health and Nutrition Examination Survey (NHANES), a cross-sectional survey of a nationally representative sample of non-institutionalized Americans. Methods: The current analysis included survey cycles where both iron-related markers and mental health-related outcomes were collected in adolescents 12 to 17 years old. Acute and serious medical conditions, acute inflammation, and abnormal birth weight led to exclusion. Linear multivariable regression analyses examined the association between ID status (defined based on the total body iron model) and (1) total Patient Health Questionnaire (PHQ-9) score, (2) one item examining anxiety severity, and (3) one item examining overall mental well-being. Covariates included age, sex, race and ethnicity, body mass index, household income, head-of-household marital status, and psychotropic medication use. Sensitivity analyses examined the robustness of the findings when ID was defined based on the ferritin model. Results: In 1990 adolescents (age [mean ± SD]: 14.5 ± 1.7 years; 85.7% females), ID with and without anemia was significantly associated with a higher PHQ-9 score in multiracial adolescents (Cohen’s d = 1.09, *p* = 0.0005 for ID without anemia; d = 0.92, *p* = 0.0395 for ID with anemia). Moreover, ID with anemia was associated with more severe anxiety (d = 3.00, *p* = 0.0130) and worse mental well-being (d = 2.75, *p* = 0.0059) in multiracial adolescents. The findings remained significant after adjusting for psychotropic use and in the sensitivity analyses. Conclusions: Iron deficiency is associated with poorer mental health in adolescents of multiracial background. Future studies should confirm these findings prospectively and examine the underlying mechanism.

## 1. Introduction

Iron is essential for neurogenesis, synaptogenesis, neurotransmitter synthesis, and myelination [1,2]. Yet, iron deficiency (ID) is the most common nutritional deficiency worldwide, disproportionately affecting infants and adolescents, particularly in minoritized groups [3,4,5]. In fact, up to 40% of American adolescent girls may have ID [6], with non-Hispanic Black and Mexican American females of childbearing age being twice as likely to be iron deficient compared to their non-Hispanic White counterparts [7]. However, ID is underdiagnosed, with most cases remaining unidentified until the emergence of symptoms or anemia [8].

The increased susceptibility of adolescents, particularly females, to ID at a time when synaptic pruning peaks and myelination is still ongoing [9,10,11] raises concerns about the neuropsychiatric sequelae of ID in this age group. In fact, ID has been associated with psychopathology [12,13,14], with depressive and anxiety disorders being twice as prevalent in children and adolescents with ID anemia, compared to those without [15]. Even in adolescents with ID but without anemia, preliminary evidence shows an increased risk for ADHD, and anxiety and depressive disorders [16,17,18,19]. However, research in this area and in this population is nascent, relying on relatively small sample sizes. In contrast, more and larger studies have been completed in adults, finding a higher prevalence of depressive symptoms in nonpregnant females with ID [20].

Given the high prevalence of ID and of depressive symptoms in adolescents [21], and taking advantage of data from the National Health and Nutrition Examination Survey (NHANES), the present study sought to examine the relationship between ID, both with and without anemia, and symptoms of depression and anxiety in adolescents. Disentangling the effects of ID from those of anemia is necessary given that the latter itself causes irritability, apathy, fatigue, low mood, and concentration difficulties, which may all be associated with depressive and anxiety disorders. We hypothesized that ID will be associated with increased symptom severity, after accounting for potential confounding variables.

## 2. Materials and Methods

### 2.1. Participants

The NHANES is a cross-sectional, nationally representative survey of the noninstitutionalized civilian population of the United States, conducted in 2-year cycles [22]. About 5000 participants are selected each survey cycle to undergo health questionnaires and examinations. Study procedures have been extensively documented elsewhere [23,24]. The current analysis focused on survey cycles where both iron-related markers (e.g., serum ferritin or sF) and mental health-related outcomes (e.g., Patient Health Questionnaire-9 or PHQ-9) were collected in adolescents 12 to 17 years old. These comprise survey cycles 2005–2006, 2007–2008, 2009–2010, 2015–2016, and 2017–2018.

Participants having any of the following were excluded from the analyses:

1. Non-iron deficiency anemia, defined as having anemia but normal body iron status, given that such conditions involve different pathophysiology, with some even causing iron overload;
2. High sensitivity C-reactive protein (hsCRP) concentration > 10 mg/dL, as this indicates acute inflammation, which may increase sF, an acute phase reactant [25]. This would confound the association between body iron status and mental health-related outcomes [25];
3. Hemoglobin concentration > 16.4 g/dL in males and >15.3 g/dL in females as these might reflect disease states such as conditions associated with hemoconcentration or heart, pulmonary, or bone marrow diseases [26];
4. Elevated sF concentration, defined as >200 ng/mL in males and >150 ng/mL in females [27], as these likely reflect diseases (e.g., auto-immune conditions) that would confound the associations of interest;
5. Chronic medical conditions or medications indicative of a serious medical condition or acute inflammation (e.g., antineoplastics or antibiotics), as they affect iron availability and may have their own mental health sequalae [28];
6. Birth weight < 2.5 or >4.0 kg, given its association with neonatal ID and poor neurodevelopmental or psychiatric outcomes [29,30].

The NHANES protocol is approved by the National Center for Health Statistics Research Ethics Review Board. As the current analyses involved deidentified data, collected for an unrelated purpose, they do not represent human subject research and no further ethics review board approval was needed [31].

### 2.2. Assessment of Body Iron Status

The following variables capturing body iron status were used: sF, which is widely used for diagnosing ID [25], total body iron (TBI, mg/kg) computed as −[log10 (sTfR× 1000/sF)-2.8229]/0.1207, where sTfR is soluble transferrin receptor concentration [32], transferrin saturation, and free erythrocyte protoporphyrin. In the primary analyses, ID was defined based on the TBI model, with negative TBI values (i.e., TBI < 0 mg/kg) indicating ID [32]. Sensitivity analyses were carried out, using alternative definitions of ID, including two definitions based on the ferritin model (with a cutoff of 15 ng/mL [25] or 30 ng/mL [33]) and one based on a more conservative, earlier definition by the Centers for Disease Control and Prevention, requiring 2 of the following 3 indicators: (1) sF < 15 ng/mL, (2) transferrin saturation < 16%, and (3) free erythrocyte protoporphyrin > 70 mcg/dL [25,34,35]. Hemoglobin was used to determine anemia status [26].

### 2.3. Assessment of Internalizing Symptoms

The primary outcome of interest was the PHQ-9 total score. The PHQ-9 is a reliable and valid screening tool for detecting depression in both clinical and research settings, including in adolescents [36,37]. It comprises 9 items, based on the symptom criteria for a depressive episode as indicated in the Diagnostic and Statistical Manual of Mental Disorders, Fifth Edition, Text Revision [38]. Each item is scored from 0 to 3 to indicate not at all, several days, more than half the days, and nearly every day, respectively. A cutoff of 10 indicates the presence of clinically significant symptoms of depression [36].

Additional outcome variables included two items from the Health Status Questionnaire capturing anxiety symptoms and overall mental health, respectively (HSQ496, “During the past 30 days, for about how many days have you felt worried, tense, or anxious?” and HSQ480, “Now thinking about your mental health, which includes stress, depression, and problems with emotions, for how many days during the past 30 days was your mental health not good?”).

### 2.4. Covariates

Covariates included age, sex, race and ethnicity, age–sex-specific body mass index Z-score (BMI), household income, head-of-household marital status, and psychotropic medication use. Age was treated as a continuous variable. Sex was a binary variable (i.e., male/female). Race and ethnicity were combined into a single variable and categorized as non-Hispanic White, non-Hispanic Black, Mexican Hispanic, non-Mexican Hispanic, and other, based on self-report. Family income was reduced to four groups: <$20,000, $20,000 to <$45,000, $45,000 to <$75,000, and ≥$75,000. Head-of-household marital status was categorized as married/living with partner, widowed/divorced/separated, and never married. Psychotropic medication use was converted to a binary variable.

### 2.5. Analysis

All analyses accounted for survey weights, included in the NHANES dataset. A sample weight is assigned to each participant to account for the multistage, probability sampling design (including oversampling), survey non-response, and post-stratification adjustment to match total population counts from the Census Bureau [22]. Missing data for BMI Z-score, marital status, and household income were imputed, using multiple imputation implemented in the R package “mice” (v3.15.0) [39].

To examine the possibility of a dose effect, participants were assigned to one of 4 groups based on their ID and anemia status: (1) iron-deficiency anemia (IDA), (2) iron deficiency without anemia (ID-Only), (3) non-iron-deficiency anemia, defined as having anemia with a normal level of iron, and (4) no iron deficiency or anemia (CON for controls). As noted earlier, ID was defined based on the TBI model in the primary analyses, with sensitivity analyses using alternative definitions. In addition, anemia with no ID led to exclusion. Anemia was defined as the presence of a hemoglobin concentration <12.4 g/dL in males and <11.4 g/dL in females [26].

To compare the different groups, analysis of variance (ANOVA) was used for continuous variables and Chi square tests or Fisher’s Exact tests were used for categorical ones. Multivariable linear regression analyses examined the association between body iron status and self-reported mental-health related variables (i.e., PHQ-9 score and items HSQ496 and HSQ480). Interaction terms of body iron status with sex and race/ethnicity were also entered. As applicable, participants without ID, those who are male, and those who are non-Hispanic White served as the reference groups. We also examined the association of body iron status with the presence of clinically significant depressive symptoms severity, as indicated by PHQ-9 score ≥10 [36], using logistic regression analyses. All analyses were run twice, with and without adjusting for psychotropic medication use.

All analyses were performed using R, version 4.2.9 (R Core Team), with 2-sided statistical test and significance defined as *p* < 0.05. Data analyses were conducted between August 2022 and April 2024. Of note, because the data related to internalizing symptoms are under restricted access, the analyses could only be carried out at designated Research Data Centers, managed by the Census Bureau, after Special Status Security clearance is granted. Moreover, to prevent any possibility of confidentiality breach, certain restrictions on data reporting applied. For instance, the minimum and maximum values of variables and sample sizes consisting of ≤5 participants could not be disclosed. As such, “N/A” will be entered in the Tables, where applicable.

## 3. Results

After excluding participants with missing data, those with non-iron-deficiency anemia, and those who failed to meet our inclusion/exclusion criteria, a total of 1990 adolescents were included in the analyses. The data of interest (i.e., internalizing symptoms and iron-related markers) were jointly available for males only in survey cycle 2017–2018. As such, the sample was composed largely of female participants (85.7%). Table 1 summarizes the demographic and clinical characteristics of all participants. Similar descriptive data are provided in Tables S1–S3, using alternative ID definitions. There were significant differences in age, sex, and racial/ethnic composition between groups of different ID and anemia statuses. Significant group differences were observed in PHQ-9 total score only when using sF < 15 ng/mL to define ID (*p* = 0.0201, Table S1) and in the number of participants having a PHQ-9 total score ≥ 10 only when using sF < 30 ng/mL to define ID (*p* = 0.0153, Table S2).

After controlling for age (*p* = 0.0327), sex (*p* < 0.0001), household income (*p* = 0.0065), head of household’s marital status (*p* < 0.005), and BMI (*p* < 0.001), neither the main effect of ID groups nor of race/ethnicity on PHQ-9 scores was significant (all *p* values > 0.14), except for that of the Other Hispanic subgroup (*p* = 0.0198). With regard to item HSQ496, neither the main effect of ID groups nor of race/ethnicity was significant (all *p* values > 0.12) after controlling for age (*p* = 0.0349), household income (*p* > 0.5), head of household’s marital status (*p* > 0.2), and BMI (*p* > 0.9). Finally, with regard to item HSQ480, neither the main effect of ID groups nor of race/ethnicity on PHQ-9 scores was significant (all *p* values > 0.09), except for that of the Non-Hispanic Black (*p* = 0.0029) and Other Hispanic (*p* = 0.0050) subgroups, after controlling for age (*p* < 0.0001), household income (*p* = 0.0074), head of household’s marital status (*p* < 0.05), and BMI (*p* = 0.0661).

After controlling for age, sex, BMI, household income, and head-of-household marital status, the interaction effect of race/ethnicity with body iron status was significant, whereby participants of other racial (including multiracial) background with ID-Only and with IDA had a higher PHQ-9 total score compared to non-Hispanic White participants (Cohen’s d = 1.09 for ID-Only and d = 0.92 for IDA; Table 2). Adjusting for psychotropic medication use did not alter the findings (Table S5).

No significant associations were observed between body iron status and depression status, using PHQ-9 as a binary outcome (Table S6).

Data for items HSQ496 and HSQ480 were only available for female participants. After controlling for age, BMI, household income, and head-of-household marital status, the interaction effect of race/ethnicity and body iron status was again significant, whereby participants of other racial background with IDA had higher scores (d = 3.00 for item HSQ496 and d = 2.75 for item HSQ480; Table 2). These effects remained significant after accounting for psychotropic medication use (Table S5).

When ID was defined based on sF < 15 ng/mL, the interaction effect of race/ethnicity and body iron status remained significant, with participants of other racial background with IDA displaying higher scores on the PHQ-9, HSQ496, and HSQ480 (Table S7). When ID was defined based on sF < 30 ng/mL, the findings remained significant for the HSQ496 and HSQ480 items in participants of other racial background with IDA (d = 2.85 for item HSQ496 and d = 2.60 for item HSQ480; Table S8). Notably, participants identifying as Mexican American with ID-Only had less days with symptoms of anxiety (d = −1.4; Table S8). The analyses using the three indicators to define ID as suggested by the CDC yielded no significant associations, though the sample size was substantially smaller (Table S9). All the other findings in the sensitivity analyses were not altered.

## 4. Discussion

To our knowledge, this is the largest study to examine the association of ID with symptoms of depression and anxiety in adolescents, along with the moderating effect of race and ethnicity. Having sF < 15 or 30 ng/mL was associated with a higher PHQ-9 score or a higher likelihood of having a PHQ-9 score > 10, respectively. However, after accounting for relevant confounders, ID without anemia was significantly associated with more severe depression only in adolescents of multiracial background as well as those who do not identify as White, Black, or Hispanic. Moreover, when anemia was present, indicating a higher degree of ID, worse anxiety and mental wellbeing were observed. The findings were comparable when using alternate definitions of ID, based on sF, and after adjusting for psychotropic use. Our findings add to the growing literature characterizing the association between ID and psychopathology, which has thus far focused mostly on young adults and women of reproductive age [20,24,40]. Another strength of our work lies in the fact that the sample was nationally representative, making our findings generalizable.

Several factors should be considered when interpreting our findings, chief among them the NHANES study design and the likely complex relationship between peripheral and brain iron levels. The NHANES is a cross-sectional study. As such, the findings reflect mental health symptom severity and body iron status at the time of data collection, with no information about their duration. In particular, the available data cannot explain why the association between ID and worse depressive symptoms was restricted to the other/multiracial group. Given that this group presumably included participants from South Asian background, one hypothesis is that individuals in this group are more likely to be vegetarian, implying a longer duration of ID, with a more extended impact on brain iron stores. Another possibility is that individuals from a multiracial background have earlier onset of menarche [41], again implying a longer duration of ID. However, no significant differences were observed between the racial/ethnic subgroups with regard to vegetarian diet, red meat consumption, or age at menarche (all *p* values > 0.10). Regardless of the mechanism underlying our novel finding, it is important to note that, in 2021, past-year prevalence of major depressive episode in multiracial adolescents in the USA was 27.2%, the highest of any racial or ethnic group [21]. This disproportionate vulnerability to depression underscores the urgent need to further explore ID as a potential contributing factor to worse mental health in adolescents. This is particularly important considering known racial/ethnic disparities in susceptibility to ID. In fact, ID is nearly three times more prevalent in Mexican American infants compared to their White American counterparts [42]. In menstruating individuals, Mexican Americans and Black Americans are twice as likely to have ID as White Americans [7,43]. Similarly, Black Americans have lower hemoglobin concentrations than Americans of other racial/ethnic subgroups [44,45]. Importantly, these differences reflect health disparities related to social determinants of health rather than inherent biological differences [46].

Iron plays a pivotal role in brain development, starting in utero. In fact, in rodents, primates, and humans, pre- and postnatal ID has been associated with impaired myelination and neurotransmitter signaling, and cognitive deficits [47,48,49]. Humans are unique in having a pubertal growth spurt in height [50]. This involves rapid growth in the size of bodily organs, requiring large amounts of nutrients, including iron, accompanied by drastic changes in brain structure and function. The increase in bodily demands for iron coincides with increasing iron loss in menstruating individuals. As such, adolescence has been associated with an elevated incidence of ID. How peripheral ID may affect brain iron stores in adolescents is not well established. Studies using animal models have shown that iron transport across the blood–brain barrier (BBB) peaks early during development [51]. Moreover, available evidence suggests the presence of critical periods during early neurodevelopment following which the long-term neurocognitive sequelae of brain iron depletion may not be reversible, despite iron supplementation [52,53,54,55]. On the other hand, postmortem tissue and non-invasive brain imaging studies in humans have shown that iron continues to accumulate in healthy brains throughout life [56,57]. Importantly, early in life, iron is diverted in a hierarchical manner to prioritize hematopoiesis [58]. This includes brain iron, which has been shown to be affected in ID [59]. This is biologically plausible given the critical need to optimize hematopoiesis for survival. If this mechanism also applies to adolescents, ID would disrupt physiological brain iron accumulation, potentially derailing normal brain development. However, this process will vary in its magnitude depending on several factors including ID severity, duration, and, critically, timing in relation to the timing of other brain developmental processes. 

The ferritin and TBI models capture the status of different body iron compartments, with the former reflecting iron stores and the latter reflecting transport iron [60]. In turn, low sF indicates ID, while low TBI indicates ID erythropoiesis. It is only when hemoglobin production is reduced below a certain cutoff that anemia manifests. Our analysis revealed more consistent associations between IDA and the mental health outcomes of interest. However, it is notable that the associations remained largely consistent when ID was defined based on the TBI versus the ferritin model. This is important for two reasons: (1) consistency in the findings adds rigor and strength to them, and (2) measuring soluble transferrin receptor following an approach compatible with the method developed by Cook et al. is not readily accessible [60]. In contrast, measuring sF is more widely available. However, the debate continues regarding the most appropriate cutoff for ID [61], with evidence mounting to suggest that the cutoff of 15 ng/mL may be too conservative, resulting in an unnecessarily high false-negative rate [62,63,64]. It has been argued that combining different indicators of iron status to define ID increases the positive predictive value but reduces the sensitivity [65]. In other words, defining ID following the CDC’s requirement of abnormalities in sF, transferrin saturation, and free erythrocyte protoporphyrin may be restrictive, leading to many individuals with ID not being identified. Our analysis found that only a small number of participants meet this conservative threshold to define ID, rendering the analysis underpowered. 

In addition to the cross-sectional design mentioned previously, additional study limitations must be mentioned. While, to our knowledge, this is the largest study to examine the association of body iron stores and mental health-related outcomes in adolescents, those with ID and IDA represented a small proportion, including among multiracial participants. Second, although we examined different approaches to defining ID, the ultimate index of interest is brain iron content, which was not measured in the NHANES. We adjusted for several covariates, but many others were unavailable to account for, including sexual maturation ratings and sleep hygiene. The latter is particularly important given the complex interaction between ID, sleep, and mental health [66,67,68,69]. Also, anxiety and overall mental wellbeing were captured using a single item, which is suboptimal, and no such data were available in male participants. In fact, overall, boys comprised only about 14% of the sample, precluding our ability to examine the impact of ID in boys more comprehensively. In addition, no data were available from other informants acquainted with the participants. Typically, parent input is key to the comprehensive assessment of children and adolescents [70].

In summary, findings from this nationally representative sample of American adolescents add to the growing body of evidence linking ID to poorer mental health outcomes. Given the high prevalence of ID in the US and worldwide, as well as that of depression and anxiety disorders, there is an urgent need for prospective studies examining the impact of ID on brain development and psychopathology, while measuring markers of brain iron content in addition to peripheral markers of body iron stores.

## Figures and Tables

**Table 1:** Demographic and clinical characteristics of the participants with complete total body iron data.

	Total *n* = 1990	ID-Only *n* = 64	IDA *n* = 57	Controls *n* = 1869	*p* Value
**TBI (mg/kg), mean ± SD**	6.3 ± 3.5	−1.4 ± 1.2	−3.8 ± 2.5	6.9 ± 2.7	**<0.0001**
**Age (years), mean ± SD**	14.5 ± 1.7	15.1 ± 1.7	15.4 ± 1.5	14.5 ± 1.7	**<0.0001**
**Sex, *n* (%)**					**0.0041**
Male	285 (14.3)	N/A ^1^	N/A ^1^	280 (15.0)	
Female	1705 (85.7)	N/A ^1^	N/A ^1^	1589 (85.0)	
**Race/ethnicity, *n* (%)**					**0.0169**
Non-Hispanic White	566 (28.4)	10 (15.6)	N/A ^1^	549 (29.4)	
Non-Hispanic Black	554 (27.8)	22 (34.4)	15 (26.3)	517 (27.7)	
Mexican American	171 (8.6)	8 (12.5)	N/A ^1^	159 (8.5)	
Other Hispanic	477 (24.0)	15 (23.4)	21 (36.8)	441 (23.6)	
Other, including Multiracial	222 (11.2)	9 (14.1)	10 (17.5)	203 (10.9)	
**BMI (kg/m^2^), mean ± SD**	24.0 ± 5.8	24.8 ± 6.7	23.7 ± 4.7	24.0 ± 5.8	0.5275
**Psychotropic medication use, *n* (%)**	108 (5.4)	N/A ^1^	N/A ^1^	104 (5.6)	0.4416
**Household income, *n* (%)**					0.2809
<$20,000 per year	651 (32.7)	16 (25.0)	17 (29.8)	618 (33.1)	
$20,000 to <$45,000 per year	438 (22.0)	14 (21.9)	17 (29.8)	407 (21.8)	
$45,000 to <$75,000 per year	372 (18.7)	17 (26.6)	9 (15.8)	346 (18.5)	
≥$75,000 per year	529 (26.6)	17 (26.6)	14 (24.6)	498 (26.6)	
**Head of household’s marital status, *n* (%)**					0.7751
Married or living with a partner	1331 (66.9)	47 (73.4)	36 (63.2)	1248 (66.8)	
Divorced, separated, or widowed	414 (20.8)	11 (17.2)	14 (24.6)	389 (20.8)	
Never married	245 (12.3)	6 (9.4)	7 (12.3)	232 (12.4)	
**Serum ferritin (ng/mL), mean ± SD**	37.7 ± 27.6	6.4 ± 1.7	5.3 ± 2.9	39.8 ± 27.2	**<0.0001**
Transferrin saturation (%), mean ± SD *	23.0 ± 11.0	11.3 ± 5.4	5.2 ± 2.2	23.8 ± 10.7	**<0.0001**
FEP (ug/dL RBC), mean ± SD **	58.2 ± 24.8	92.2 ± 29.3	139.3 ± 46.8	54.9 ± 19.4	**<0.0001**
**PHQ-9 Score**	3.2 ± 3.8	3.5 ± 4.2	4.1 ± 4.4	3.2 ± 3.8	0.2021
**PHQ-9 score >10, *n* (%)**	149 (7.5)	N/A ^1^	7 (12.3)	138 (7.4)	0.3568
HSQ496 Item (days), mean ± SD ***	1.8 ± 2.1	1.8 ± 2.0	2.0 ± 2.2	1.8 ± 2.1	0.9191
HSQ480 Item (days), mean ± SD ****	3.4 ± 6.3	4.2 ± 6.7	3.0 ± 5.2	3.4 ± 6.3	0.6814

ID-Only: iron deficiency without anemia, whereby total body iron or TBI is < 0 mg/kg but hemoglobin concentration is ≥ 12.4 g/dL in males and ≥ 11.4 g/dL in females; IDA: iron deficiency with anemia, whereby TBI is < 0 mg/kg and hemoglobin concentration is < 12.4 g/dL in males and < 11.4 g/dL in females. BMI: body mass index; FEP: free erythrocyte protoporphyrin; PHQ-9: Patient Health Questionnaire-9; RBC: red blood cells; TBI: total body iron. * missing values, *n* = 1077. ** missing values, *n* = 510. HSQ496 item: “During the past 30 days, for about how many days have you felt worried, tense, or anxious?”. *** missing values, *n* = 596. HSQ480 item: “Now thinking about your mental health, which includes stress, depression, and problems with emotions, for how many days during the past 30 days was your mental health not good?”. **** missing values, *n* = 1111. ^1^ N/A: Data are withheld when sample size is < 5 by Census Bureau rules (see analysis section). **Bolded** *p* values are significant (*p* < 0.05).

**Table 2:** Results of the multivariable regression analyses examining the main and interaction effects of race/ethnicity and iron status (based on total body iron) in predicting indicators of mental health.

	β	SE	*p* Value	Cohen’s d
**PHQ-9 score**				
**Main effects**				
ID-Only	−2.1	1.2	0.0829	
IDA	−0.4	1.4	0.7811	
Non-Hispanic Black	−0.4	0.3	0.1385	
Mexican American	−0.3	0.4	0.3846	
Other Hispanic	−**0.6**	**0.3**	**0.0406**	
Other or Multiracial	−0.3	0.3	0.3869	
**Results for the interaction effect of race/ethnicity and ID-Only status**				
Non-Hispanic Black	2.8	1.4	0.0514	0.20
Mexican AmericanOther Hispanic	2.3	1.8	0.1982	0.07
	0.4	1.5	0.8007	−0.45
Other or Multiracial	**6.1**	**1.7**	**0.0005**	**1.09**
**Results for the interaction effect of race/ethnicity and IDA status**				
Non-Hispanic Black	1.1	1.7	0.5284	0.19
Mexican American	2.8	2.4	0.2402	0.64
Other Hispanic	−0.02	1.6	0.9917	−0.11
Other or Multiracial	**3.8**	**1.9**	**0.0395**	**0.92**
**HSQ496 Item**				
**Main effects**				
ID-Only	0.4	1.0	0.6762	
IDA	−0.2	1.5	0.8763	
Non-Hispanic Black	−0.3	0.4	0.4119	
Mexican American	−0.5	0.4	0.2364	
Other Hispanic	−0.4	0.3	0.2869	
Other or Multiracial	−0.1	0.4	0.7202	
**Results for the interaction effect of race/ethnicity and ID-Only status**				
Non-Hispanic Black	−0.6	1.2	0.6340	−0.08
Mexican American	−1.6	1.4	0.2681	−0.55
Other Hispanic	2.4	1.6	0.1314	1.38
Other or Multiracial	−1.7	1.6	0.2817	−0.64
**Results for the interaction effect of race/ethnicity and IDA status**				
Non-Hispanic Black	0.04	1.7	0.9814	−0.09
Mexican American	N/A ^1^	N/A ^1^	N/A ^1^	N/A ^1^
Other Hispanic	−0.1	1.7	0.9474	−0.16
Other or Multiracial	**6.3**	**2.5**	**0.0130**	**3.00**
**HSQ480 Item**				
**Main effects**				
ID-Only	−1.7	2.5	0.5049	
IDA	−2.2	3.1	0.4759	
Non-Hispanic Black	**−2.5**	**0.8**	**0.0018**	
Mexican American	−1.3	0.9	0.1588	
Other Hispanic	**−2.1**	**0.8**	**0.0063**	
Other or Multiracial	−0.7	0.9	0.4364	
**Results for the interaction effect of race/ethnicity and ID-Only status**				
Non-Hispanic Black	4.1	3.0	0.1702	0.39
Mexican American	−2.0	3.8	0.6071	−0.59
Other Hispanic	2.7	3.0	0.3646	0.17
Other or Multiracial	3.1	4.4	0.4853	0.23
**Results for the interaction effect of race/ethnicity and IDA status**				
Non-Hispanic Black	3.3	3.7	0.3715	0.18
Mexican American	N/A ^1^	N/A ^1^	N/A ^1^	N/A ^1^
Other Hispanic	0.3	3.6	0.9274	−0.31
Other or Multiracial	**19.1**	**6.9**	**0.0059**	**2.75**

ID-Only: iron deficiency without anemia, whereby total body iron or TBI is <0 mg/kg but hemoglobin concentration is ≥12.4 g/dL in males and ≥11.4 g/dL in females; IDA: iron deficiency with anemia, whereby TBI is <0 mg/kg and hemoglobin concentration is <12.4 g/dL in males and <11.4 g/dL in females. PHQ-9: Patient Health Questionnaire-9; SE: standard error. HSQ496 item: “During the past 30 days, for about how many days have you felt worried, tense, or anxious?”. HSQ480 item: “Now thinking about your mental health, which includes stress, depression, and problems with emotions, for how many days during the past 30 days was your mental health not good?”. ^1^ N/A: not available. Data are withheld when sample size is <5 by Census Bureau rules (see analysis section). Empty cells reflect data that cannot be calculated. **Bolded** effects are significant (*p* < 0.05). Participants without ID served as the reference group for the other ID groups and Non-Hispanic White participants served as the reference group for the other racial/ethnic groups.

## Data Availability

The datasets presented in this article are the property of the NCHS, with some of the data being publicly available while others under restricted use. Investigators interested in the restricted data must apply for access through the NCHS (https://www.cdc.gov/nchs/tutorials/nhanes-cms/orientation/accessing.htm, accessed on 20 August 2024).

## Supplementary Materials

The following supporting information can be downloaded at: https://www.mdpi.com/article/10.3390/nu16213643/s1, Table S1: Demographic and clinical characteristics of the participants when using serum ferritin concentration <15 ng/mL to define iron deficiency; Table S2: Demographic and clinical characteristics of the participants when using serum ferritin concentration <30 ng/mL to define iron deficiency; Table S3: Demographic and clinical characteristics of the participants when using serum ferritin concentration, transferrin saturation, and free erythrocyte protoporphyrin concentration to define iron deficiency; Table S4: Results of the multivariable regression analyses examining the interaction effect of sex and iron status in predicting PHQ-9 score; Table S5: Results of the multivariable regression analyses examining the interaction effect of race/ethnicity and iron status in predicting indicators of mental health, with use of psychotropic medications as an additional covariate; Table S6: Results of the logistic regression analyses examining the interaction effect of race/ethnicity and iron status in predicting having a PHQ-9 score ≥ 10, using different definitions of iron deficiency; Table S7: Results of the multivariable regression analyses examining the interaction effect of race/ethnicity and iron status in predicting indicators of mental health when using serum ferritin concentration <15 ng/mL to define iron deficiency; Table S8: Results of the multivariable regression analyses examining the interaction effect of race/ethnicity and iron status in predicting indicators of mental health when using serum ferritin concentration < 30 ng/mL to define iron deficiency; Table S9: Results of the multivariable regression analyses examining the interaction effect of race/ethnicity and iron status in predicting indicators of mental health when using serum ferritin concentration, transferrin saturation, and free erythrocyte protoporphyrin concentration to define iron deficiency.
