# Supplementary material for: Iron Deficiency and Internalizing Symptoms Among Adolescents in the National Health and Nutrition Examination Survey
Source: Nutrients. 2024 Oct 26;16(21):3643. doi: 10.3390/nu16213643 (PMC11547248; doi:10.3390/nu16213643)
Supplement: Supplementary file 1 [file nutrients-16-03643-s001.zip › nutrients-3194832-supplementary.pdf]

**Iron Deficiency and Internalizing Symptoms Among Adolescents in the National Health and Nutrition Examination Survey.**

**Supplementary Materials**

**Table S1.** Demographic and clinical characteristics of the participants when using serum ferritin concentration < 15 ng/mL to define iron deficiency.

| | **Total** *n* **= 2021** | **ID-Only** *n* **= 265** | **IDA** *n* **= 76** | **Controls** *n* **= 1680** | ***p* Value** |
|---|---|---|---|---|---|
| **Age (years), mean ± SD** | 14.6 ± 1.7 | 15.0 ± 1.6 | 15.4 ± 1.4 | 14.4 ± 1.7 | **<0.0001** |
| **Sex, n (%)** | | | | | **<0.0001** |
| Male | 286 (14.2) | 9 (3.4) | N/A[1] | 273 (16.3) | |
| Female | 1735 (85.8) | 256 (96.6) | N/A[1] | 1407 (83.8) | |
| **Race/ethnicity, n (%)** | | | | | **0.0106** |
| Non-Hispanic White | 576 (28.5) | 68 (25.7) | 13 (17.1) | 495 (29.5) | |
| Non-Hispanic Black | 558 (27.6) | 86 (32.5) | N/A[1] | 455 (27.1) | |
| Mexican American | 173 (8.6) | 27 (10.2) | N/A[1] | 142 (8.5) | |
| Other Hispanic | 489 (24.2) | 55 (20.8) | 30 (39.5) | 404 (24.0) | |
| Other, including Multiracial | 225 (11.1) | 29 (10.9) | 12 (15.8) | 184 (11.0) | |
| **BMI (kg/m$^2$), mean ± SD** | 24.0 ± 5.8 | 24.2 ± 5.8 | 23.8 ± 4.8 | 24.0 ± 5.8 | 0.8211 |
| **Psychotropic medication use, n (%)** | 110 (5.4) | 13 (4.9) | N/A[1] | 95 (5.7) | 0.4814 |
| **Household income, n (%)** | | | | | 0.9546 |
| < $20,000 per year | 662 (32.8) | 88 (33.21) | 28 (36.84) | 546 (32.50) | |
| $20,000 to < $45,000 per year | 452 (22.4) | 55 (20.75) | 18 (23.68) | 379 (22.56) | |
| $45,000 to < $75,000 per year | 375 (18.6) | 53 (20.00) | 12 (15.79) | 310 (18.45) | |
| ≥ $75,000 per year | 532 (26.3) | 69 (26.04) | 18 (23.68) | 445 (26.49) | |
| **Head of household's marital status, n (%)** | | | | | 0.5794 |
| Married or living with a partner | 1354 (67.0) | 187 (70.6) | 49 (64.5) | 1118 (66.5) | |
| Divorced, separated, or widowed | 420 (20.8) | 51 (19.2) | 19 (25) | 350 (20.1) | |
| Never married | 247 (12.2) | 27 (10.2) | 8 (10.5) | 212 (12.6) | |
| **PHQ-9 Score** | 3.2 ± 3.8 | 3.7 ± 4.3 | 3.8 ± 4.3 | 3.1 ± 3.7 | **0.0201** |
| **PHQ-9 score >10, n (%)** | 152 (7.5) | 25 (9.4) | 9 (11.8) | 118 (7.0) | 0.1332 |
| **HSQ496 Item (days), mean ± SD*** | 1.8 ± 2.1 | 1.9 ± 2.1 | 2.1 ± 2.4 | 1.8 ± 2.0 | 0.7141 |
| **HSQ480 Item (days), mean ± SD**** | 3.4 ± 6.3 | 3.9 ± 7.3 | 2.7 ± 4.9 | 3.3 ± 6.1 | 0.4067 |

ID-Only: iron deficiency without anemia, whereby serum ferritin concentration or sF is < 15 ng/mL but hemoglobin concentration is ⩾ 12.4 g/dL in males and ⩾ 11.4 g/dL in females; IDA: iron deficiency with anemia, whereby sF is < 15 ng/mL and hemoglobin concentration is < 12.4 g/dL in males and < 11.4 g/dL in females.

BMI: body mass index; PHQ-9: Patient Health Questionnaire-9; TBI: total body iron.

HSQ496 item: "During the past 30 days, for about how many days have you felt worried, tense, or anxious?"

*missing values, *n* = 484

HSQ480 item: "Now thinking about your mental health, which includes stress, depression, and problems with emotions, for how many days during the past 30 days was your mental health not good?"

**missing values, *n* = 922

[1]N/A: Data are withheld when sample size is < 5 by Census Bureau rules (see analysis section).

**Bolded** *p* values are significant ($p < 0.05$).

**Table S2.** Demographic and clinical characteristics of the participants when using serum ferritin concentration < 30 ng/mL to define iron deficiency.

| | **Total** $n$ **= 2040** | **ID-Only** $n$ **= 894** | **IDA** $n$ **= 95** | **Controls** $n$ **= 1051** | $p$ **Value** |
|---|---|---|---|---|---|
| **Age (years), mean ± SD** | 14.5 ± 1.7 | 15.0 ± 1.7 | 15.2 ± 1.5 | 14.4 ± 1.7 | **0.0001** |
| **Sex, n (%)** | | | | | **<0.0001** |
| Male | 288 (14.1) | 52 (5.8) | 6 (6.3) | 230 (21.9) | |
| Female | 1752 (85.9) | 842 (94.2) | 89 (93.7) | 821 (78.1) | |
| **Race/ethnicity, n (%)** | | | | | **<0.0001** |
| Non-Hispanic White | 578 (28.3) | 236 (26.4) | 15 (15.8) | 327 (31.1) | |
| Non-Hispanic Black | 560 (27.5) | 280 (31.3) | 19 (20) | 261 (24.8) | |
| Mexican American | 174 (8.5) | 86 (9.6) | 5 (5.3) | 83 (7.9) | |
| Other Hispanic | 503 (24.7) | 204 (22.8) | 44 (46.3) | 255 (24.3) | |
| Other, including Multiracial | 225 (11.0) | 88 (9.8) | 12 (12.6) | 125 (11.9) | |
| **BMI (kg/m²), mean ± SD** | 24.0 ± 5.8 | 23.8 ± 5.5 | 24.3 ± 5.5 | 24.2 ± 6.0 | 0.2325 |
| **Psychotropic medication use, n (%)** | 110 (5.4) | 42 (4.7) | N/A[1] | 66 (6.3) | 0.1065 |
| **Household income, n (%)** | | | | | 0.5105 |
| < $20,000 per year | 668 (32.7) | 296 (33.11) | 34 (35.79) | 338 (32.16) | |
| $20,000 to < $45,000 per year | 454 (22.3) | 202 (22.60) | 24 (25.26) | 228 (21.69) | |
| $45,000 to < $75,000 per year | 380 (18.6) | 178 (19.91) | 16 (16.84) | 186 (17.70) | |
| ≥ $75,000 per year | 538 (26.4) | 218 (24.38) | 21 (22.11) | 299 (28.45) | |
| **Head of household's marital status, n (%)** | | | | | 0.5974 |
| Married or living with a partner | 1359 (66.6) | 602 (67.3) | 56 (58.9) | 701 (66.7) | |
| Divorced, separated, or widowed | 427 (20.9) | 183 (20.5) | 25 (26.3) | 219 (20.8) | |
| Never married | 254 (12.5) | 109 (12.2) | 14 (14.7) | 131 (12.5) | |
| **PHQ-9 Score** | 3.2 ± 3.8 | 3.4 ± 4.0 | 3.7 ± 4.1 | 3.1 ± 3.6 | 0.0833 |
| **PHQ-9 score >10, n (%)** | 154 (7.5) | 80 (8.9) | 11 (11.6) | 63 (6.0) | **0.0153** |
| **HSQ496 Item (days), mean ± SD*** | 1.8 ± 2.1 | 1.9 ± 2.1 | 2.2 ± 2.2 | 1.7 ± 2.0 | 0.3552 |
| **HSQ480 Item (days), mean ± SD**** | 3.4 ± 6.3 | 3.4 ± 6.5 | 2.4 ± 4.4 | 3.4 ± 6.2 | 0.6143 |

ID-Only: iron deficiency without anemia, whereby serum ferritin concentration or sF is < 30 ng/mL but hemoglobin concentration is ⩾ 12.4 g/dL in males and ⩾ 11.4 g/dL in females; IDA: iron deficiency with anemia, whereby sF is < 30 ng/mL and hemoglobin concentration is < 12.4 g/dL in males and < 11.4 g/dL in females.

BMI: body mass index; PHQ-9: Patient Health Questionnaire-9; TBI: total body iron.

HSQ496 item: "During the past 30 days, for about how many days have you felt worried, tense, or anxious?"

*missing values, $n$ = 285

HSQ480 item: "Now thinking about your mental health, which includes stress, depression, and problems with emotions, for how many days during the past 30 days was your mental health not good?"

**missing values, $n$ = 548

[1]N/A: Data are withheld when sample size is < 5 by Census Bureau rules (see analysis section).

**Bolded** $p$ values are significant ($p < 0.05$).

**Table S3.** Demographic and clinical characteristics of the participants when using serum ferritin concentration, transferrin saturation, and free erythrocyte protoporphyrin concentration to define iron deficiency*.

| | Total<br>*n* = 506 | ID-Only<br>*n* = 365 | IDA<br>*n* = 6 | Controls<br>*n* = 135 | *p* Value |
|---|---|---|---|---|---|
| **Age (years), mean ± SD** | 14.5 ± 1.7 | 14.5 ± 1.7 | 14.8 ± 2.0 | 14.5 ± 1.8 | 0.8930 |
| **Sex, n (%)** | | | | | N/A |
| Male | 0 (0.0) | 0 (0.0) | 0 (0.0) | 0 (0.0) | |
| Female | 506 (100.0) | 365 (100.0) | 6 (100.0) | 135 (100.0) | |
| **Race/ethnicity, n (%)** | | | | | **0.0022** |
| Non-Hispanic White | 120 (23.7) | 104 (28.5) | N/A[1] | 61 (45.2) | |
| Non-Hispanic Black | 184 (36.4) | 122 (33.4) | N/A[1] | N/A | |
| Mexican American | 14 (2.8) | 11 (3.0) | N/A[1] | 49 (36.3) | |
| Other Hispanic | 164 (32.4) | 110 (30.1) | N/A[1] | 6 (4.4) | |
| Other, including Multiracial | 24 (4.7) | 18 (4.9) | N/A[1] | N/A | |
| **BMI (kg/m$^2$), mean ± SD** | 23.9 ± 5.8 | 23.3 ± 5.3 | | 25.5 ± 6.4 | **0.0007** |
| **Psychotropic medication use, n (%)** | 18 (3.6) | 13 (3.6) | N/A[1] | 5 (3.7) | 0.8915 |
| **Household income, n (%)** | | | | | 0.5109 |
| < $20,000 per year | 158 (31.2) | 120 (32.88) | N/A[1] | 35 (25.93) | |
| $20,000 to < $45,000 per year | 122 (24.1) | 90 (24.66) | N/A[1] | 31 (22.96) | |
| $45,000 to < $75,000 per year | 111 (21.9) | 76 (20.82) | N/A[1] | 34 (25.19) | |
| ≥ $75,000 per year | 115 (22.7) | 79 (21.64) | N/A[1] | 35 (25.93) | |
| **Head of household's marital status, n (%)** | | | | | 0.2611 |
| Married or living with a partner | 326 (64.4) | 239 (65.5) | N/A[1] | 85 (63.0) | |
| Divorced, separated, or widowed | 117 (23.1) | 86 (23.6) | N/A[1] | 29 (21.5) | |
| Never married | 63 (12.5) | 40 (11.0) | N/A[1] | 21 (15.6) | |
| **PHQ-9 Score** | 2.9 ± 3.4 | 2.8 ± 3.4 | 1.0 ± 0.6 | 3.0 ± 3.3 | 0.3382 |
| **PHQ-9 score >10, n (%)** | 30 (5.9) | 22 (6.0) | N/A[1] | 8 (5.9) | 0.8251 |
| **HSQ480 Item (days), mean ± SD** | 3.0 ± 6.2 | 2.9 ± 6.2 | 0.8 ± 2.0 | 3.3 ± 6.3 | 0.6007 |

*This definition of iron deficiency suggested by the Centers for Disease Control and Prevention requires 2 of the following 3 indicators: 1) sF < 15 ng/mL, 2) transferrin saturation < 16%, and 3) free erythrocyte protoporphyrin > 70 mcg/dL.

ID-Only: iron deficiency without anemia; IDA: iron deficiency with anemia.

BMI: body mass index; PHQ-9: Patient Health Questionnaire-9; TBI: total body iron.

HSQ496 item: "During the past 30 days, for about how many days have you felt worried, tense, or anxious?"

HSQ480 item: "Now thinking about your mental health, which includes stress, depression, and problems with emotions, for how many days during the past 30 days was your mental health not good?"

[1]N/A: Data are withheld when sample size is < 5 by Census Bureau rules (see analysis section).

**Bolded** *p* values are significant ($p < 0.05$).

**Table S4.** Results of the multivariable regression analyses examining the interaction effect of sex and iron status in predicting PHQ-9 score.

| Definition of iron deficiency used | β | SE | *p* Value | Cohen's d |
|---|---|---|---|---|
| **TBI < 0** | | | | |
| **Main effects** | | | | |
| ID-Only | 1.6 | 2.7 | 0.5445 | |
| IDA | 0.2 | 2.2 | 0.9116 | |
| Female sex | **1.2** | **0.2** | **<0.0001** | |
| **Results for the interaction effect of sex and iron status** | | | | |
| ID-Only | -1.6 | 2.7 | 0.5696 | 0.02 |
| IDA | 0.5 | 2.2 | 0.8147 | 0.21 |
| **sF < 15 ng/mL** | | | | |
| **Main effects** | | | | |
| ID-Only | 1.4 | 1.3 | 0.2852 | |
| IDA | 1.4 | 1.9 | 0.4542 | |
| Female sex | **1.1** | **0.3** | **<0.0001** | |
| **Results for the interaction effect of sex and iron status** | | | | |
| ID-Only | -1.0 | 1.3 | 0.4558 | 0.10 |
| IDA | -0.9 | 1.9 | 0.6461 | 0.14 |
| **sF < 30 ng/mL** | | | | |
| **Main effects** | | | | |
| ID-Only | 0.4 | 0.6 | 0.4864 | |
| IDA | 0.9 | 1.5 | 0.5491 | |
| Female sex | **1.2** | **0.3** | **<0.0001** | |
| **Results for the interaction effect of sex and iron status** | | | | |
| ID-Only | -0.3 | 0.6 | 0.6426 | 0.03 |
| IDA | -0.5 | 1.6 | 0.7432 | 0.11 |

ID-Only: iron deficiency without anemia; IDA: iron deficiency with anemia; PHQ-9: Patient Health Questionnaire-9; SE: standard error; sF: serum ferritin concentration; TBI: total body iron.

**Bolded** effects are significant ($p < 0.05$).

Participants without ID served as the reference group for the other ID groups and male participants served as the reference group for females.

**Table S5.** Results of the multivariable regression analyses examining the interaction effect of race/ethnicity and iron status in predicting indicators of mental health, with use of psychotropic medications as an additional covariate.

| Definition of iron deficiency used | β | SE | *p* Value | Cohen's d |
|---|---|---|---|---|
| **PHQ-9 score** | | | | |
| **Main effects** | | | | |
| ID-Only | -2.1 | 1.2 | 0.0787 | |
| IDA | -0.2 | 1.4 | 0.9041 | |
| Non-Hispanic Black | -0.2 | 0.3 | 0.4340 | |
| Mexican American | -0.07 | 0.4 | 0.8487 | |
| Other Hispanic | -0.4 | 0.3 | 0.2074 | |
| Other or Multiracial | -0.1 | 0.3 | 0.7446 | |
| **Results for the interaction effect of race/ethnicity and ID-Only status** | | | | |
| Non-Hispanic Black | 2.7 | 1.4 | 0.0613 | 0.16 |
| Mexican American | 2.3 | 1.8 | 0.1880 | 0.07 |
| Other Hispanic | 0.4 | 1.5 | 0.7728 | -0.44 |
| Other or Multiracial | **6.2** | **1.7** | **0.0003** | **1.13** |
| **Results for the interaction effect of race/ethnicity and IDA status** | | | | |
| Non-Hispanic Black | 0.9 | 1.7 | 0.5843 | 0.21 |
| Mexican American | 2.6 | 2.3 | 0.2676 | 0.66 |
| Other Hispanic | -0.3 | 1.6 | 0.8511 | -0.13 |
| Other or Multiracial | **3.7** | **1.8** | **0.0433** | **0.97** |
| **HSQ496 Item** | | | | |
| **Main effects** | | | | |
| ID-Only | 0.5 | 1.0 | 0.6182 | |
| IDA | -0.3 | 1.5 | 0.8572 | |
| Non-Hispanic Black | -0.3 | 0.4 | 0.3583 | |
| Mexican American | -0.5 | 0.4 | 0.1950 | |
| Other Hispanic | -0.4 | 0.3 | 0.2418 | |
| Other or Multiracial | -0.2 | 0.4 | 0.6406 | |
| **Results for the interaction effect of race/ethnicity and ID-Only status** | | | | |
| Non-Hispanic Black | -0.7 | 1.3 | 0.5780 | -0.09 |
| Mexican American | -1.6 | 1.4 | 0.2424 | -0.55 |
| Other Hispanic | 2.3 | 1.6 | 0.1453 | 1.39 |
| Other or Multiracial | -1.8 | 1.6 | 0.2587 | -0.64 |
| **Results for the interaction effect of race/ethnicity and IDA status** | | | | |
| Non-Hispanic Black | 0.06 | 1.7 | 0.9716 | -0.10 |
| Mexican American | N/A[1] | N/A[1] | N/A[1] | N/A[1] |
| Other Hispanic | -0.08 | 1.7 | 0.9619 | -0.17 |
| Other or Multiracial | **6.4** | **2.5** | **0.0124** | **3.00** |
| **HSQ480 Item** | | | | |
| **Main effects** | | | | |
| ID-Only | -2.0 | 2.5 | 0.4259 | |
| IDA | -1.9 | 3.1 | 0.5303 | |
| Non-Hispanic Black | **-2.2** | **0.8** | **0.0049** | |
| Mexican American | -1.0 | 0.9 | 0.2711 | |
| Other Hispanic | **-1.9** | **0.8** | **0.0156** | |

| | | | | |
|---|---|---|---|---|
| Other or Multiracial | -0.5 | 0.9 | 0.6091 | |
| **Results for the interaction effect of race/ethnicity and ID-Only status** | | | | |
| Non-Hispanic Black | 4.0 | 2.9 | 0.1692 | 0.33 |
| Mexican American | -1.6 | 3.8 | 0.6759 | -0.59 |
| Other Hispanic | 3.1 | 3.0 | 0.2953 | 0.19 |
| Other or Multiracial | 3.5 | 4.4 | 0.4306 | 0.24 |
| **Results for the interaction effect of race/ethnicity and IDA status** | | | | |
| Non-Hispanic Black | 3.1 | 3.7 | 0.3981 | 0.20 |
| Mexican American | N/A[1] | N/A[1] | N/A[1] | N/A[1] |
| Other Hispanic | -0.2 | 3.6 | 0.9609 | -0.35 |
| Other or Multiracial | **1.9** | **6.9** | **0.0062** | **2.78** |

ID-Only: iron deficiency without anemia, whereby total body iron or TBI is < 0 mg/kg but hemoglobin concentration is ≥ 12.4 g/dL in males and ≥ 11.4 g/dL in females; IDA: iron deficiency with anemia, whereby TBI is < 0 mg/kg and hemoglobin concentration is < 12.4 g/dL in males and < 11.4 g/dL in females.
BMI: body mass index; PHQ-9: Patient Health Questionnaire-9; TBI: total body iron.
HSQ496 item: "During the past 30 days, for about how many days have you felt worried, tense, or anxious?"
HSQ480 item: "Now thinking about your mental health, which includes stress, depression, and problems with emotions, for how many days during the past 30 days was your mental health not good?"
[1]N/A: Data are withheld when sample size is < 5 by Census Bureau rules (see analysis section).
**Bolded** effects are significant ($p < 0.05$).
Participants without ID served as the reference group for the other ID groups and Non-Hispanic White participants served as the reference group for the other racial/ethnic groups.

**Table S6.** Results of the logistic regression analysis examining the interaction effect of race/ethnicity and iron status in predicting having a PHQ-9 score ≥ 10, using different definitions of iron deficiency.

| Definition of iron deficiency used | OR | 95% CI | *p* Value |
|---|---|---|---|
| **TBI < 0** | | | |
| **Main effects** | | | |
| ID-Only | <0.01 | 0, ∞ | 0.9849 |
| IDA | 2.16 | 0.25, 18.73 | 0.4831 |
| Non-Hispanic Black | 0.85 | 0.48, 1.53 | 0.6041 |
| Mexican American | 0.54 | 0.25, 1.23 | 0.1459 |
| Other Hispanic | **0.47** | **0.25, 0.89** | **0.0196** |
| Other or Multiracial | 0.61 | 0.28, 1.32 | 0.2098 |
| **Results for the interaction effect of race/ethnicity and ID-Only status** | | | |
| Non-Hispanic Black | $16x10^6$ | 0, ∞ | 0.9848 |
| Mexican American | 1.63 | 0, ∞ | 0.9997 |
| Other Hispanic | 1.12 | 0, ∞ | 0.9999 |
| Other or Multiracial | $68x10^6$ | 0, ∞ | 0.9833 |
| **Results for the interaction effect of race/ethnicity and IDA status** | | | |
| Non-Hispanic Black | 0.31 | 0.02, 6.14 | 0.4432 |
| Mexican American | 2.00 | 0.08, 49.77 | 0.6716 |
| Other Hispanic | 0.65 | 0.05, 9.19 | 0.7511 |
| Other or Multiracial | 2.00 | 0.12, 32.09 | 0.6257 |
| **sF < 15 ng/mL** | | | |
| **Main effects** | | | |
| ID-Only | 1.08 | 0.45, 2.56 | 0.8651 |
| IDA | 1.93 | 0.40, 9.30 | 0.4111 |
| Non-Hispanic Black | 0.85 | 0.47, 1.55 | 0.6009 |
| Mexican American | 0.51 | 0.21, 1.25 | 0.1406 |
| Other Hispanic | **0.48** | **0.25, 0.92** | **0.0281** |
| Other or Multiracial | 0.72 | 0.33, 1.57 | 0.4081 |
| **Results for the interaction effect of race/ethnicity and ID-Only status** | | | |
| Non-Hispanic Black | 1.44 | 0.46, 4.53 | 0.5298 |
| Mexican American | 2.03 | 0.38, 10.86 | 0.4060 |
| Other Hispanic | 0.72 | 0.16, 3.30 | 0.6755 |
| Other or Multiracial | 1.02 | 0.17, 6.28 | 0.9770 |
| **Results for the interaction effect of race/ethnicity and IDA status** | | | |
| Non-Hispanic Black | 0.69 | 0.08, 6.17 | 0.7426 |
| Mexican American | 2.60 | 0.15, 46.17 | 0.5143 |
| Other Hispanic | 0.55 | 0.06, 4.85 | 0.5925 |
| Other or Multiracial | 1.68 | 0.17, 17.13 | 0.6590 |
| **sF < 30 ng/mL** | | | |
| **Main effects** | | | |
| ID-Only | 1.01 | 0.57, 1.94 | 0.8627 |
| IDA | 2.85 | 0.73, 11.16 | 0.1320 |
| Non-Hispanic Black | 0.73 | 0.35, 1.53 | 0.4011 |
| Mexican American | 0.25 | 0.05, 1.10 | 0.0666 |
| Other Hispanic | **0.41** | **0.19, 0.92** | **0.0324** |
| Other or Multiracial | 0.70 | 0.27, 1.84 | 0.4723 |

| | | | |
|---|---|---|---|
| **Results for the interaction effect of race/ethnicity and ID-Only status** | | | |
| Non-Hispanic Black | 1.51 | 0.63, 3.62 | 0.3561 |
| Mexican American | 3.94 | 0.72, 21.67 | 0.1150 |
| Other Hispanic | 1.35 | 0.50, 3.64 | 0.5566 |
| Other or Multiracial | 1.13 | 0.30, 4.28 | 0.8535 |
| **Results for the interaction effect of race/ethnicity and IDA status** | | | |
| Non-Hispanic Black | 0.50 | 0.06, 3.90 | 0.5051 |
| Mexican American | 3.35 | 0.17, 64.94 | 0.4236 |
| Other Hispanic | 0.43 | 0.06, 2.87 | 0.3835 |
| Other or Multiracial | 1.24 | 0.13, 11.69 | 0.8499 |
| **Definition based on three biomarkers*** | | | |
| **Main effects** | | | |
| ID-Only | 1.09 | 0.12, 10.13 | 0.9373 |
| IDA | 0.85 | 0, ∞ | 0.9954 |
| Non-Hispanic Black | 9.07 | 0.26, 316.25 | 0.2235 |
| Mexican American | 0.87 | 0, ∞ | 0.9967 |
| Other Hispanic | 4.77 | 0.14, 159.71 | 0.3829 |
| Other or Multiracial | <0.01 | 0, ∞ | 0.9946 |
| **Results for the interaction effect of race/ethnicity and ID-Only status** | | | |
| Non-Hispanic Black | 0.83 | 0.06, 10.93 | 0.8865 |
| Mexican American | $19 \times 10^{6}$ | 0, ∞ | 0.9967 |
| Other Hispanic | 1.11 | 0.08, 15.61 | 0.9397 |
| Other or Multiracial | 2.02 | 0, ∞ | 0.9980 |
| **Results for the interaction effect of race/ethnicity and IDA status** | | | |
| Non-Hispanic Black | 1.70 | 0, ∞ | 0.9999 |
| Mexican American | N/A[1] | N/A[1] | N/A[1] |
| Other Hispanic | N/A[1] | N/A[1] | N/A[1] |
| Other or Multiracial | N/A[1] | N/A[1] | N/A[1] |

*This definition of iron deficiency suggested by the Centers for Disease Control and Prevention requires 2 of the following 3 indicators: 1) sF < 15 ng/mL, 2) transferrin saturation < 16%, and 3) free erythrocyte protoporphyrin > 70 mcg/dL.

ID-Only: iron deficiency without anemia; IDA: iron deficiency with anemia.

BMI: body mass index; CI: confidence interval; OR: odds ratio; PHQ-9: Patient Health Questionnaire-9; sF: serum ferritin concentration; TBI: total body iron.

[1]N/A: Data are withheld when sample size is < 5 by Census Bureau rules (see analysis section).

**Bolded** effects are significant ($p < 0.05$).

Participants without ID served as the reference group for the other ID groups and Non-Hispanic White participants served as the reference group for the other racial/ethnic groups.

**Table S7.** Results of the multivariable regression analyses examining the interaction effect of race/ethnicity and iron status in predicting indicators of mental health when using serum ferritin concentration < 15 ng/mL to define iron deficiency.

| | β | SE | *p* Value | Cohen's d |
|---|---|---|---|---|
| **PHQ-9 score** | | | | |
| **Main effects** | | | | |
| ID-Only | 0.8 | 0.5 | 0.0993 | 0.22 |
| IDA | -0.2 | 1.1 | 0.8734 | -0.04 |
| Non-Hispanic Black | -0.3 | 0.3 | 0.3204 | |
| Mexican American | -0.1 | 0.4 | 0.7308 | |
| Other Hispanic | -0.6 | 0.3 | 0.0702 | |
| Other or Multiracial | -0.06 | 0.4 | 0.8653 | |
| **Results for the interaction effect of race/ethnicity and ID-Only status** | | | | |
| Non-Hispanic Black | -0.4 | 0.7 | 0.5396 | 0.11 |
| Mexican American | -0.7 | 0.9 | 0.4349 | 0.02 |
| Other Hispanic | -0.6 | 0.7 | 0.4240 | 0.06 |
| Other or Multiracial | -0.4 | 0.9 | 0.6451 | 0.11 |
| **Results for the interaction effect of race/ethnicity and IDA status** | | | | |
| Non-Hispanic Black | 1.1 | 1.4 | 0.4374 | 0.25 |
| Mexican American | 2.5 | 2.2 | 0.2422 | 0.63 |
| Other Hispanic | -0.5 | 1.3 | 0.6961 | -0.18 |
| Other or Multiracial | **3.5** | **1.5** | **0.0224** | **0.89** |
| **HSQ496 Item** | | | | |
| **Main effects** | | | | |
| ID-Only | 0.3 | 0.4 | 0.5293 | 0.12 |
| IDA | -0.2 | 1.5 | 0.8985 | -0.09 |
| Non-Hispanic Black | -0.2 | 0.4 | 0.5624 | |
| Mexican American | -0.5 | 0.4 | 0.2737 | |
| Other Hispanic | -0.4 | 0.4 | 0.2203 | |
| Other or Multiracial | -0.1 | 0.4 | 0.7952 | |
| **Results for the interaction effect of race/ethnicity and ID-Only status** | | | | |
| Non-Hispanic Black | -0.6 | 0.6 | 0.3199 | -0.16 |
| Mexican American | -0.6 | 0.8 | 0.3999 | -0.19 |
| Other Hispanic | 0.8 | 0.6 | 0.2426 | 0.49 |
| Other or Multiracial | -0.9 | 1.0 | 0.3581 | -0.31 |
| **Results for the interaction effect of race/ethnicity and IDA status** | | | | |
| Non-Hispanic Black | -0.5 | 1.7 | 0.7687 | -0.34 |
| Mexican American | N/A[1] | N/A[1] | N/A[1] | N/A[1] |
| Other Hispanic | 0.5 | 1.6 | 0.7586 | 0.15 |
| Other or Multiracial | **6.3** | **2.6** | **0.0146** | **2.97** |
| **HSQ480 Item** | | | | |
| **Main effects** | | | | |
| ID-Only | -0.2 | 1.1 | 0.8619 | -0.03 |
| IDA | -3.0 | 2.5 | 0.2417 | -0.49 |
| Non-Hispanic Black | **-2.5** | **0.8** | **0.0020** | |
| Mexican American | -1.6 | 0.9 | 0.0986 | |
| Other Hispanic | **-2.2** | **0.8** | **0.0050** | |

| | | | | |
|---|---|---|---|---|
| Other or Multiracial | -1.0 | 1.0 | 0.3300 | |
| **Results for the interaction effect of race/ethnicity and ID-Only status** | | | | |
| Non-Hispanic Black | 1.0 | 1.4 | 0.4975 | 0.13 |
| Mexican American | 0.4 | 2.1 | 0.8686 | 0.03 |
| Other Hispanic | 0.9 | 1.5 | 0.5178 | 0.12 |
| Other or Multiracial | 1.6 | 2.3 | 0.4793 | 0.24 |
| **Results for the interaction effect of race/ethnicity and IDA status** | | | | |
| Non-Hispanic Black | 3.9 | 3.3 | 0.2376 | 0.15 |
| Mexican American | N/A[1] | N/A[1] | N/A[1] | N/A[1] |
| Other Hispanic | 1.1 | 3.0 | 0.7115 | -0.30 |
| Other or Multiracial | **0.2** | **6.7** | **0.0028** | **2.79** |

ID-Only: iron deficiency without anemia, whereby serum ferritin concentration or sF is < 15 ng/mL but hemoglobin concentration is ≥ 12.4 g/dL in males and ≥ 11.4 g/dL in females; IDA: iron deficiency with anemia, whereby sF is < 15 ng/mL and hemoglobin concentration is < 12.4 g/dL in males and < 11.4 g/dL in females.
BMI: body mass index; PHQ-9: Patient Health Questionnaire-9; TBI: total body iron.
HSQ496 item: "During the past 30 days, for about how many days have you felt worried, tense, or anxious?"
HSQ480 item: "Now thinking about your mental health, which includes stress, depression, and problems with emotions, for how many days during the past 30 days was your mental health not good?"
[1]N/A: Data are withheld when sample size is < 5 by Census Bureau rules (see analysis section).
**Bolded** effects are significant ($p < 0.05$).
Participants without ID served as the reference group for the other ID groups and Non-Hispanic White participants served as the reference group for the other racial/ethnic groups.

**Table S8.** Results of the multivariable regression analyses examining the interaction effect of race/ethnicity and iron status in predicting indicators of mental health when using serum ferritin concentration < 30 ng/mL to define iron deficiency.

| | β | SE | *p* Value | Cohen's d |
|---|---|---|---|---|
| **PHQ-9 score** | | | | |
| **Main effects** | | | | |
| ID-Only | 0.3 | 0.3 | 0.3401 | 0.08 |
| IDA | 0.6 | 1.0 | 0.5414 | 0.16 |
| Non-Hispanic Black | -0.1 | 0.4 | 0.7098 | |
| Mexican American | -0.01 | 0.5 | 0.9796 | |
| Other Hispanic | -0.7 | 0.4 | 0.0639 | |
| Other or Multiracial | <0.01 | 0.4 | 0.9999 | |
| **Results for the interaction effect of race/ethnicity and ID-Only status** | | | | |
| Non-Hispanic Black | -0.4 | 0.5 | 0.3232 | -0.04 |
| Mexican American | -0.5 | 0.7 | 0.4809 | -0.04 |
| Other Hispanic | 0.1 | 0.5 | 0.7921 | 0.12 |
| Other or Multiracial | -0.2 | 0.6 | 0.7498 | 0.03 |
| **Results for the interaction effect of race/ethnicity and IDA status** | | | | |
| Non-Hispanic Black | 0.06 | 1.3 | 0.9632 | 0.18 |
| Mexican American | 1.3 | 2.0 | 0.5174 | 0.51 |
| Other Hispanic | -1.2 | 1.2 | 0.2880 | -0.17 |
| Other or Multiracial | 2.7 | 1.5 | 0.0708 | 0.89 |
| **HSQ496 Item** | | | | |
| **Main effects** | | | | |
| ID-Only | 0.4 | 0.3 | 0.1483 | 0.22 |
| IDA | 0.7 | 1.2 | 0.5664 | 0.34 |
| Non-Hispanic Black | -0.1 | 0.4 | 0.8117 | |
| Mexican American | 0.1 | 0.5 | 0.7725 | |
| Other Hispanic | -0.5 | 0.4 | 0.2062 | |
| Other or Multiracial | 0.3 | 0.5 | 0.5142 | |
| **Results for the interaction effect of race/ethnicity and ID-Only status** | | | | |
| Non-Hispanic Black | -0.4 | 0.4 | 0.3266 | 0.005 |
| Mexican American | **-1.4** | **0.6** | **0.0154** | **-0.46** |
| Other Hispanic | 0.4 | 0.5 | 0.3916 | 0.42 |
| Other or Multiracial | -1.3 | 0.8 | 0.0752 | -0.44 |
| **Results for the interaction effect of race/ethnicity and IDA status** | | | | |
| Non-Hispanic Black | -0.7 | 1.4 | 0.6353 | 0.006 |
| Mexican American | -0.6 | 2.4 | 0.8011 | 0.04 |
| Other Hispanic | -0.3 | 1.4 | 0.8284 | 0.19 |
| Other or Multiracial | **5.1** | **2.4** | **0.0349** | **2.85** |
| **HSQ480 Item** | | | | |
| **Main effects** | | | | |
| ID-Only | 0.5 | 0.7 | 0.4876 | 0.08 |
| IDA | -1.6 | 2.4 | 0.4871 | -0.27 |
| Non-Hispanic Black | **-1.9** | **0.9** | **0.0300** | |
| Mexican American | -0.6 | 1.1 | 0.5838 | |
| Other Hispanic | **-2.4** | **0.9** | **0.0069** | |

| | | | | |
|---|---|---|---|---|
| Other or Multiracial | 0.6 | 1.2 | 0.6564 | |
| **Results for the interaction effect of race/ethnicity and ID-Only status** | | | | |
| Non-Hispanic Black | -0.9 | 1.0 | 0.3774 | -0.06 |
| Mexican American | -1.9 | 1.5 | 0.1889 | -0.24 |
| Other Hispanic | 0.6 | 1.0 | 0.5731 | 0.17 |
| Other or Multiracial | -2.4 | 1.7 | 0.1665 | -0.31 |
| **Results for the interaction effect of race/ethnicity and IDA status** | | | | |
| Non-Hispanic Black | 1.9 | 3.0 | 0.5234 | 0.05 |
| Mexican American | 0.9 | 6.6 | 0.8859 | -0.11 |
| Other Hispanic | -0.4 | 2.7 | 0.8834 | -0.33 |
| Other or Multiracial | **0.2** | **6.7** | **0.0087** | **2.60** |

ID-Only: iron deficiency without anemia, whereby serum ferritin concentration or sF is < 30 ng/mL but hemoglobin concentration is ≥ 12.4 g/dL in males and ≥ 11.4 g/dL in females; IDA: iron deficiency with anemia, whereby sF is < 30 ng/mL and hemoglobin concentration is < 12.4 g/dL in males and < 11.4 g/dL in females.

BMI: body mass index; PHQ-9: Patient Health Questionnaire-9; TBI: total body iron.

HSQ496 item: "During the past 30 days, for about how many days have you felt worried, tense, or anxious?"

HSQ480 item: "Now thinking about your mental health, which includes stress, depression, and problems with emotions, for how many days during the past 30 days was your mental health not good?"

[1]N/A: Data are withheld when sample size is < 5 by Census Bureau rules (see analysis section).

**Bolded** effects are significant ($p < 0.05$).

Participants without ID served as the reference group for the other ID groups and Non-Hispanic White participants served as the reference group for the other racial/ethnic groups.

**Table S9.** Results of the multivariable regression analyses examining the interaction effect of race/ethnicity and iron status in predicting indicators of mental health when using serum ferritin concentration, transferrin saturation, and free erythrocyte protoporphyrin concentration to define iron deficiency*.

| | β | SE | *p* Value | Cohen's d |
|---|---|---|---|---|
| **PHQ-9 score** | | | | |
| **Main effects** | | | | |
| ID-Only | 0.4 | 0.9 | 0.6405 | 0.13 |
| IDA | -2.6 | 1.6 | 0.0976 | -0.78 |
| Non-Hispanic Black | 1.5 | 1.3 | 0.2214 | |
| Mexican American | 1.9 | 2.2 | 0.3894 | |
| Other Hispanic | 1.4 | 1.3 | 0.2728 | |
| Other or Multiracial | 0.6 | 1.6 | 0.7319 | |
| **Results for the interaction effect of race/ethnicity and ID-Only status** | | | | |
| Non-Hispanic Black | -0.9 | 1.1 | 0.3873 | -0.15 |
| Mexican American | -0.9 | 2.4 | 0.7050 | -0.14 |
| Other Hispanic | -0.7 | 1.1 | 0.5392 | -0.07 |
| Other or Multiracial | -0.8 | 1.8 | 0.6638 | -0.11 |
| **Results for the interaction effect of race/ethnicity and IDA status** | | | | |
| Non-Hispanic Black | 1.8 | 3.8 | 0.6308 | -0.24 |
| Mexican American | N/A[1] | N/A[1] | N/A[1] | N/A[1] |
| Other Hispanic | N/A[1] | N/A[1] | N/A[1] | N/A[1] |
| Other or Multiracial | N/A[1] | N/A[1] | N/A[1] | N/A[1] |
| **HSQ480 Item** | | | | |
| **Main effects** | | | | |
| ID-Only | 1.0 | 1.6 | 0.5541 | 0.13 |
| IDA | -4.5 | 2.9 | 0.1157 | -0.78 |
| Non-Hispanic Black | 0.2 | 0.2 | 0.9257 | |
| Mexican American | -0.4 | 0.4 | 0.9188 | |
| Other Hispanic | 0.9 | 0.2 | 0.7036 | |
| Other or Multiracial | -0.5 | 0.3 | 0.8752 | |
| **Results for the interaction effect of race/ethnicity and ID-Only status** | | | | |
| Non-Hispanic Black | -1.8 | 1.9 | 0.3531 | -0.15 |
| Mexican American | 1.1 | 4.3 | 0.7984 | -0.14 |
| Other Hispanic | -2.0 | 2.0 | 0.3147 | -0.07 |
| Other or Multiracial | -0.05 | 3.3 | 0.9884 | -0.11 |
| **Results for the interaction effect of race/ethnicity and IDA status** | | | | |
| Non-Hispanic Black | 7.0 | 6.8 | 0.3091 | -0.24 |
| Mexican American | N/A[1] | N/A[1] | N/A[1] | N/A[1] |
| Other Hispanic | N/A[1] | N/A[1] | N/A[1] | N/A[1] |
| Other or Multiracial | N/A[1] | N/A[1] | N/A[1] | N/A[1] |

*This definition of iron deficiency requires 2 of the following 3 indicators: 1) sF < 15 ng/mL, 2) transferrin saturation < 16%, and 3) free erythrocyte protoporphyrin > 70 mcg/dL.
ID-Only: iron deficiency without anemia; IDA: iron deficiency with anemia.
BMI: body mass index; PHQ-9: Patient Health Questionnaire-9; TBI: total body iron.
HSQ480 item: "Now thinking about your mental health, which includes stress, depression, and problems with emotions, for how many days during the past 30 days was your mental health not good?"
[1]N/A: Data are withheld when sample size is < 5 by Census Bureau rules (see analysis section).
**Bolded** effects are significant ($p < 0.05$).

Participants without ID served as the reference group for the other ID groups and Non-Hispanic White participants served as the reference group for the other racial/ethnic groups.
